# Identification of the key proteins associated with different hair types in sheep and goats

**DOI:** 10.3389/fgene.2022.993192

**Published:** 2022-09-23

**Authors:** Chongyan Zhang, Qing Qin, Zhichen Liu, Xiaolong Xu, Mingxi Lan, Yuchun Xie, Zhixin Wang, Jinquan Li, Zhihong Liu

**Affiliations:** ^1^ College of Animal Science, Inner Mongolia Agricultural University, Hohhot, China; ^2^ Key Laboratory of Mutton Sheep Genetics and Breeding of Ministry of Agriculture, Hohhot, China; ^3^ The Inner Mongolia Autonomous Region Goat Genetics and Breeding Engineering Technology Research Center, Hohhot, China; ^4^ Key Laboratory of Animal Genetics, Breeding and Reproduction in Inner Mongolia Autonomous Region, Hohhot, China

**Keywords:** cashmere, sheep, proteomics, KRT, KRTAP

## Abstract

Animal-derived fiber has the characteristics of being light, soft, strong, elastic and a good thermal insulator, and it is widely used in many industries and traditional products, so it plays an important role in the economy of some countries. Variations in phenotypes of wool fibers among different species and breeds are important for industry. We found that the mean fiber diameter of cashmere was significantly smaller than that of sheep wool (*p* < 0.01), and sheep wool was significantly smaller than goat wool (*p* < 0.01). Compared with traditional proteomics technology, we analyzed cashmere, guard hair, and wool by Laber-free proteomics technology and detected 159, 204, and 70 proteins, respectively. Through the sequential windowed acquisition of all theoretical fragmentations (SWATH), 41 and 54 differentially expressed proteins were successfully detected in the cashmere vs. wool group and guard hair vs. wool group. Protein‒protein interaction network analysis of differentially expressed proteins revealed many strong interactions related to KRT85, KRTAP15-1 and KRTAP3-1. The final analysis showed that the proportion of KRT85, KRTAP15-1 and KRTAP3-1 might be the key to the difference in fiber diameter and could be used as a potential molecular marker for distinguishing different fiber types.

## Introduction

Small ruminants, especially native breed kinds, have an vital role in the livelihood of a significant portion of human population in the developing and under developing countries (van Asten et al., 2009; Masoudzadeh et al., 2020; Mohamadipoor Saadatabadi et al., 2021). Therefore, combined trials emphasizing administration and genetic progress are decisive to improve animal production (Mohammadabadi et al., 2017). Sheep production enterprises economic and biological efficiency usually by enhance the productivity of animals and breeding to improve performance (Amiri Roudbar et al., 2017; Ghotbaldini et al., 2019). Animal fibers are valued and important commodities for textile industries worldwide and are widely used by the textile industry (Plowman et al., 2020). The highest value fibers include cashmere from goats (*Capra hircus*) and wool from sheep (*Ovis aries*). The Inner Mongolian cashmere goat is an important source of germplasm with high economic value (Wu et al., 2020b). Cashmere is finer, stronger, lighter, and softer than wool, and it looks like a luxury fiber (Dai et al., 2019). Historically, wool played an important role in the economy of Portugal and Spain in the 15th century and Britain, Australia, and New Zealand since the beginning of the industrial revolution in the 18th century (Plowman et al., 2020). Mean fiber diameter (MFD) is the most valuable fiber trait that can be measured. To a large extent, the MFD controls producers’ financial returns (Miao et al., 2018; Wang et al., 2019). Structurally, in wool and cashmere fibers, keratin (KRT) and keratin-associated proteins (KRTAP) are known as the main principal proteins. KRT and KRTAP are major structural proteins that jointly determine the quality of hair fibers in sheep and goats and the quality of general hair in mammals. In recent years, the increasing development of proteomics has also been widely used in the study of cashmere protein composition (Plowman et al., 2019). Many studies in this area have been published. These include the proteome of the wool cuticle (Koehn et al., 2010), the effect of nutrition on the proteome of merino wool (Almeida et al., 2014), and the morphogenesis of wool fiber (Plowman et al., 2015). Most proteins are identified in the keratin family, which is the key family for understanding the structure and characteristics of the fiber. As with traditional protein research methods, such as 2DE gel electrophoresis analysis and protein extraction, proteins with low abundance cannot be identified. In this respect, the information obtained by proteomics is of great significance in sequenced and studied species, including sheep (Almeida et al., 2015; Almeida et al., 2016). Plowmanet first described the wool proteome and its application in wool quality research (Plowman et al., 2000). Label-free quantification, a new quantitative proteomics technology, is usually used to identify and quantify large-scale proteins. The difference in protein expression between samples can be obtained by comparing the response signals of specific peptides/proteins in different samples. Common methods are information-dependent acquisition (IDA) and sequential windowed acquisition of all theoretical fragment ions (SWATH). SWATH does not need to label the peptides before sample analysis, which avoids the possible loss of samples. The number of detected peptides is large, the protein coverage is large, and the analysis flux is high and is not limited by the source and number of samples. The epigenome, which is composed of different mechanisms, such as DNA methylation, remodeling, histone tail modification, chromatin microRNAs and long non-coding RNAs, interacts with environmental factors like nutrition, pathogens and climate to influence gene expression profiles and the emergence of specific phenotypes (Barazandeh et al., 2019; Masoudzadeh et al., 2020). Interactions between the genome, epigenome and environmental factors may occur at multiple levels (Mohamadipoor Saadatabadi et al., 2021). In addition, there is a large body of evidence on the effects of epigenomic variation on health and productivity (Mohammadabadi et al., 2017; Barazandeh et al., 2019). Eukaryotic gene expression is temporally and multidimensional controlled (Shahsavari et al., 2021). In each tissue type, only a relatively small set of whole genomes is expressed, and the expression of genes depends on the stage of development (Mohammadabadi et al., 2021b). Thus, eukaryotic gene expression is specific to each tissue (Mohammadabadi et al., 2021a). Similarly, the amount of gene product produced in the same tissue, as well as in other tissues, regulates the expression of the gene. Studying genes and proteins is one of the basic activities of domestic animals.

In this study, the fiber types of sheep and goats were analyzed by label-free quantitative technology, aiming to explore the key proteins affecting the average fiber diameter of sheep and goats and lay the foundation for improving the quality of sheep and goats.

## Materials and methodsEthical statement

Samples were collected in accordance with the Guidelines for Experimental Animals of the Ministry of Science and Technology (Beijing, China) and were approved by the experimental animal ethics committee of Inner Mongolia Agricultural University (GB 14925-2001).

### Animals

Forty-eight animals of two sheep breeds and 62 individuals of two goat breeds were collected from the original seed farms in Inner Mongolia and Xinjiang, including 25 Xinjiang fine-wool sheep, 23 Chinese Merino fine wool sheep, 26 Albas cashmere goats, and 36 Alashan cashmere goats (female,two years old, carcass weight of 25–26 kg). The experimental animals are healthy without any disease. The detailed information is shown in Table 1. The test samples were cut from sheep and goats to measure the position of the scapula and stored in a −20° refrigerator for subsequent experiments.

**TABLE 1:** Fiber sample information.

Breed	Abbreviation	Sample size	Breed characteristics	Location
Alpas				
Cashmere	Alpas	26	cashmere and meat combination	Ordos, Inner Mongolia, China
Alashan Cashmere	Alashan	36	cashmere and meat combination	Alexa Left Banner, Inner Mongolia, China
Xinjiang fine-wool sheep	Xinjiang	25	both meat and fur	Xinjiang Seed Farm, China
Germany Merino Sheep	Merino	23	both meat and fur	Xinjiang Seed Farm, China

### Measurement of important economic characteristics

The average fiber diameter of cashmere, guard hair, and wool was measured; the structure of fiber under different magnifications was observed by scanning electron microscopy. The samples were washed and dried according to the conventional washing process and determined at constant temperature and humidity (20 ± 2°C, 65% ± 4%). An OFDA2148 fiber fineness instrument was used according to GB/T21030-2007 (wool and other animal fiber average diameter and distribution test method fiber diameter optical analyzer method). EXCEL was used for data processing, SPSS 25.0 was used for single-factor analysis of variance, and *p* < 0.05 indicated a significant difference.

### Protein extraction and digestion

The cashmere and guard hair samples of six individuals in Alpas and Alashan and six wool samples in Fine and Merino were randomly selected. A total of 0.5 g of each sample was weighed and rinsed with water. The sample was placed into a 1.5 ml centrifuge tube, 1 ml dichloromethane and methanol mixture was added, and the bed was shaken at 50°C for 2 h. The sample was centrifuged at 1,000 rpm and 4°C for 10 min, and then 1 ml of pure water was added. The sample was centrifuged again at 1,000 rpm and 4°C for 15 min, and then the sample was removed and dried at 50°C for 20 min. The samples were put into pyrolysis buffer (protease inhibitor (Roche, Basel, Switzerland) and 1% sodium dodecyl sulfate (Kulabor, China)) and put into an oven for 48 h. The samples were removed and centrifuged at 1,000 rpm and 4°C for 20 min. The supernatant was collected for further study, and the protein concentration was measured with a bicinchoninic acid (BCA) kit (Tiangen, China).

One hundred micrograms of protein was added to 200 µl of 8 M urea (Sigma, Germany) and 10 mM DL-dithiothreitol (Sigma‒Aldrich, Germany) and incubated at 37°C for 1 h. After centrifugation at 12,000 rpm for 40 min, 200 µl of urea was added to each filtrate tube, which was then agitated. Next, the filtrate tubes were centrifuged twice at 12,000 rpm for 30 min each. Then, 200 µl of 50 mM iodoacetamide (Sigma‒Aldrich, Germany) was added to each filtrate tube; the reaction was allowed to proceed in the dark for 30 min, and then the liquid was removed. Next, 100 µl of ammonium bicarbonate (Fluka, Germany) was added to each filtrate tube, and the samples were centrifuged at 12,000 rpm for 20 min. This step was performed 3 times, and then the liquid was removed. The samples were incubated overnight with trypsin at 37°C and centrifuged at 12,000 rpm for 30 min. Then, 50 µl of ammonium bicarbonate (Fluka, Germany) was added to each filtrate tube. The samples were centrifuged at 12,000 rpm for 30 min, and this step was repeated. The filtrate was collected, freeze-dried, and stored at −20°C (Wu et al., 2019).

### HPLC‒MS/MS analysis

On the LC‒MS/MS system (Sciex, Framingham, MA, United States), two methods are used, namely, the unmarked quantitative method of information-dependent acquisition (IDA) mode and the sequential window acquisition of all theoretical fragments ion spectra (SWATH). Take about 2 μg of peptide and put it on C18 HPLC column (75 μm × 15 cm).

A linear gradient (120 min, going from 5 to 80% B at 500 nL/min) of 0.1% formic acid in water and 0.1% formic acid in acetonitrile was used to separate peptides.

The conditions for IDA were as follows: resolution: 30,000; Time of flight (TOF) -ms acquisition range: 350–1800 m/z; Scanning range of automatic collision energy between MS/MS with IDA: 400–1800 m/z; The conditions of SWATH mass spectrometry are follows: MS1 collection range: 150–1,200 m/z; MS2 quality range: 100–1,500 m/z.

### Data processing

Protein Pilot 4.5 software (Sciex, Framingham, MA, United States) was used with the UniProt/SWISS-PROT/*Capra hircus* database (downloaded from https://www.UniProt.org; 556,388 proteins) to identify peptides. The results were filtered at a 1% FDR. The selected search parameters included the use of trypsin as the enzyme, allowing up to two missed cleavage sites. The peptide mass tolerance was ±15 ppm, and the fragment mass tolerance was 20 mmu. The data were loaded into PeakView (Sciex, Framingham, MA, United States) software to search the SWATH databank using the ion library generated in Protein Pilot. PeakView generated extracted ion chromatograms (XICs) after processing targeted and nontargeted data. Then, the results were interpreted and quantitatively analyzed using MarkerView software (Sciex, Framingham, MA, United States). MarkerView allows a rapid review of data to determine the DEPs. Volcano plot analysis, which combined fold change analysis and t tests, were performed. A fold change >2 or fold change <0.5 and statistical significance (*p* value <0.05) were used to identify DEPs (Ebhardt et al., 2017).

### Determination of RNA sequence

Skin samples from three animals (female,two years old, carcass weight of 25–26 kg), all of which were of Inner Mongolia cashmere goat (Alpas, Yiwei White cashmere Goat Breeding FarmErdos, Inner Mongolia). Total RNA was extracted from skin tissue using TRIzol (Takara, Dalian, China). Then, according to the manufacturer’s instructions, the samples were ground in liquid nitrogen. The concentration and purity of RNA were detected using a Beckman DU 800 Nucleic Acid-Protein Analyzer (Beckman-Coulter, Fullerton, CA, United States). The integrity of the total RNA was analyzed by 1% agarose gel electrophoresis. In addition, the extracted RNA was synthesized into cDNA (Illumina TruSeq™ RNA Sample Preparation Kit, United States) for transcriptome sequencing. Next-generation cDNA sequencing was performed using the Illumina HiSeq2000 platform.

## Results

### Determination and analysis of sheep and goat fiber traits

**FIGURE 1:**
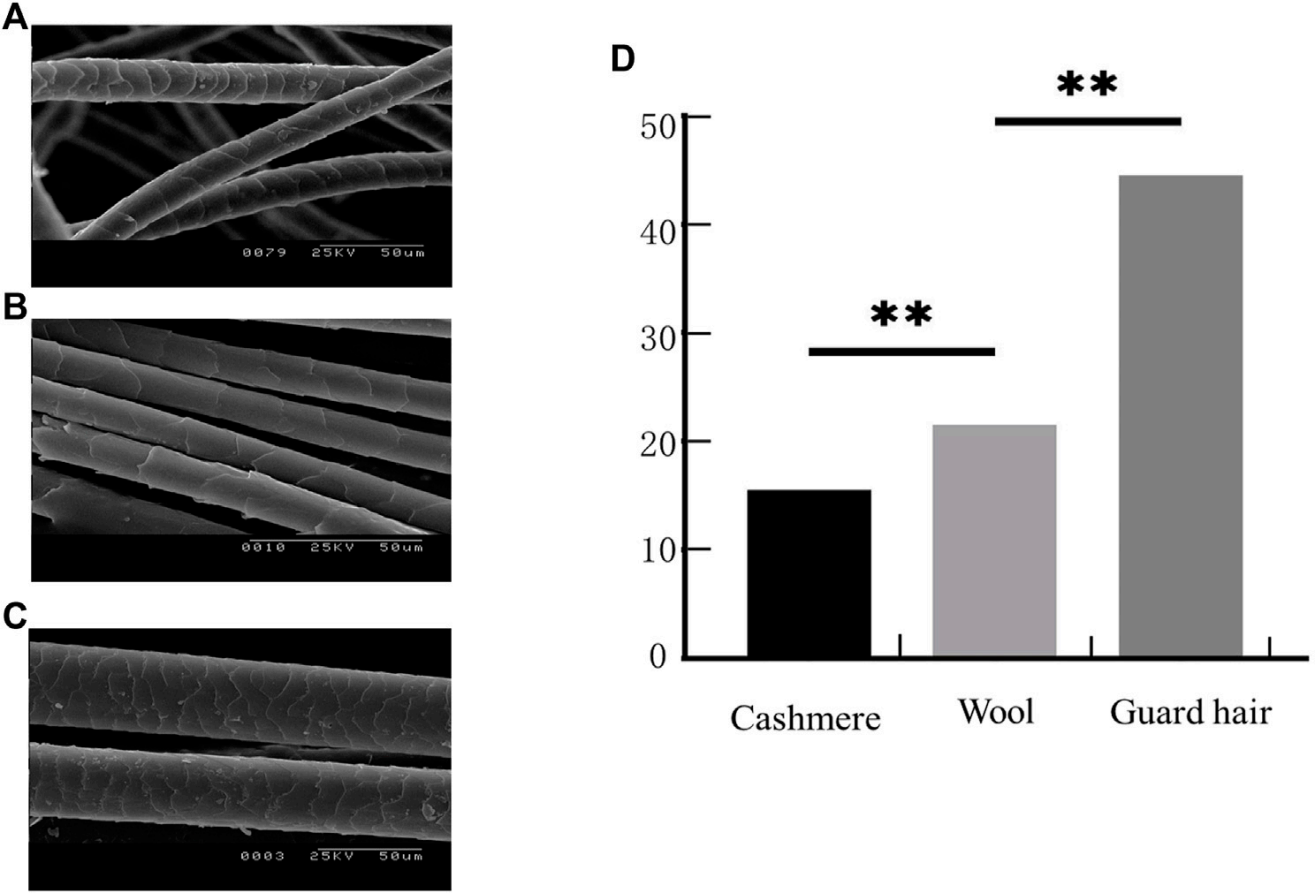
Fiber phenotypic traits. **(A)**. Sheep fiber scale structure. **(B)**. Cashmere fiber scale structure. **(C)**. Goat fiber scale structure. **(D)**. Three types of fiber fineness values (T test: **p < 0.01).

The microstructure of the fiber samples was observed. At the scale height, cashmere was greater than wool, and wool was greater than guard hair (Figure 1A–C). The average fiber diameters of the Alpas, Alashan, Fine, and Merino samples were measured (Table 2). Cashmere was significantly smaller than wool (*p* < 0.01), and wool was significantly smaller than guard hair (*p* < 0.01) (Figure 1D).

**TABLE 2:** Determination of fiber diameter.

Category		Mean fiber diameter/um±SD
Alpas cashmere	Cashmere	16.1515 ± 0.5451
	Guard hair	45.5427 ± 7.2777
Alashan cashmere	Cashmere	14.6372 ± 1.1655
	Guard hair	42.7856 ± 5.8736
Xinjiang fine-wool sheep	Wool	19.7828 ± 1.1822
Merino	Wool	23.1770 ± 1.7617
Cashmere vs wool		<0.001
Guard hair vs wool		>0.001

### Analysis of protein expression in cashmere and wool

**FIGURE 2:**
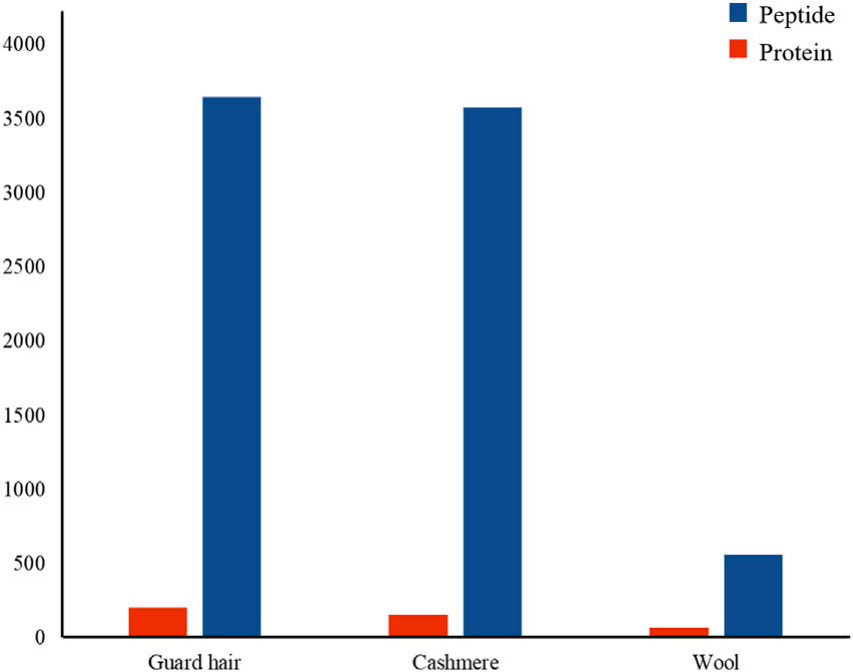
Protein identification and analysis in the hair fibers of sheep and goats.

To understand the protein composition of sheep and goat fibers, unmarked mass spectrometry was used to study the white matter based on the UniProt/SwissProt/Capra-hircus database. With a false discovery rate (FDR) ≤ 0.01, we found that 226 proteins, including 221 well-characterized proteins, were shared in both sheep and goat fibers in the present study. Of these 221 annotated proteins, 69 (31%) were keratin or keratin-associated proteins. In total, over 3,578 unique peptides and 159 proteins were quantified in cashmere. Of these 159 annotated proteins, 44 (28%) were keratin and 26 (16%) were keratin-associated proteins. Over 3,648 unique peptides and 204 proteins were quantified in guard hair, including 49 (24%) keratin and 23 (11%) keratin-associated proteins; over 563 unique peptides and 70 proteins were quantified in wool, including 29 (41%) keratin and 8 (11%) keratin-associated proteins (Figure 2). There are differences in the number of protein groups between sheep and goat fibers, so there are many proteins in sheep and goat fibers to be studied.

### Identification of proteins with different abundance in hair fibers

**FIGURE 3:**
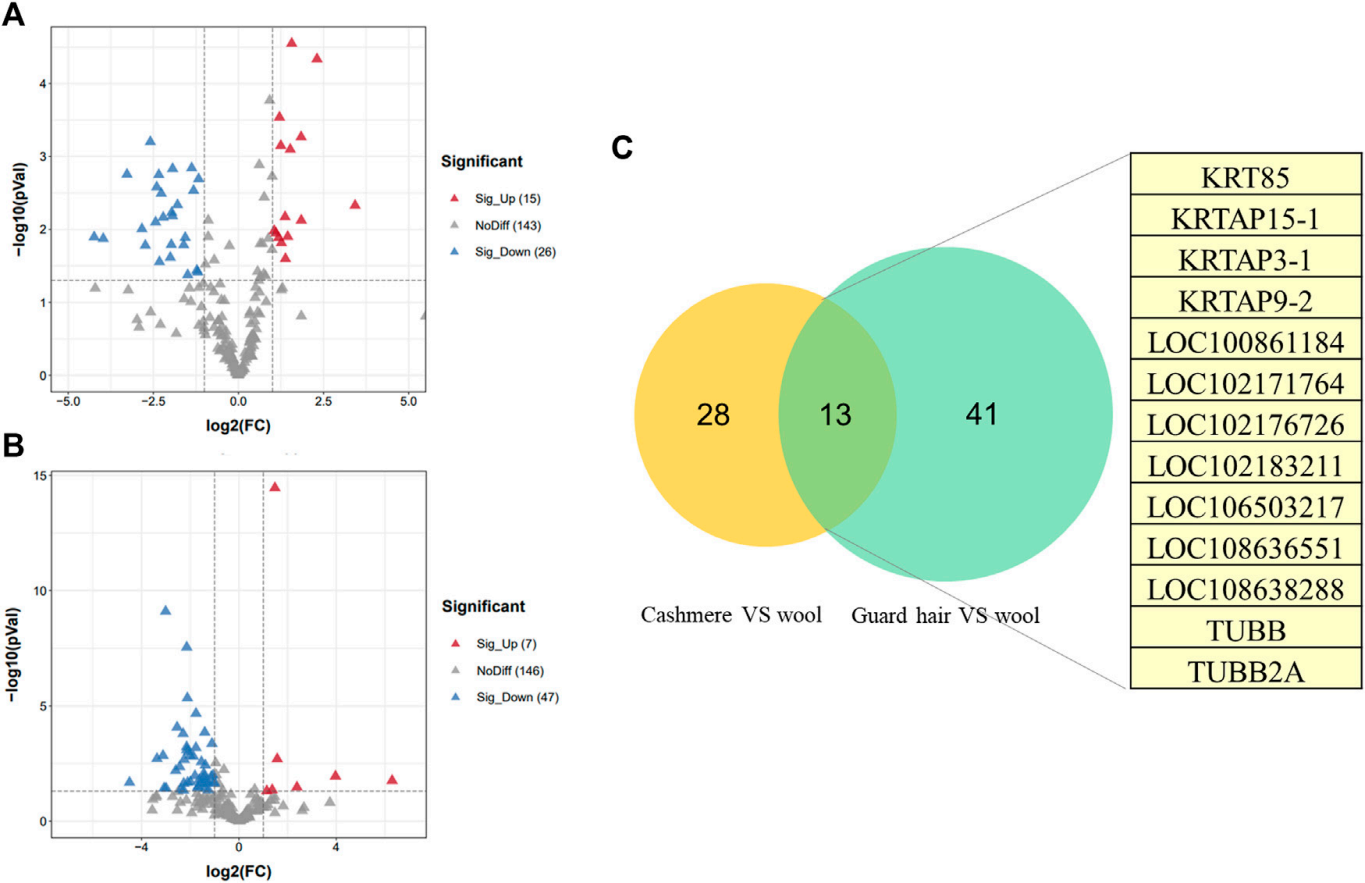
Differentially expressed proteins (DEPs) between sheep and goats. **(A)**. Volcano plot of distribution trends for DEPs between cashmere and wool. **(B)**. Volcano plot of distribution trends for DEPs between guard hair and wool. **(C)**. The Venn diagram shows differentially expressed proteins in common between the two groups in this study.

**FIGURE 4:**
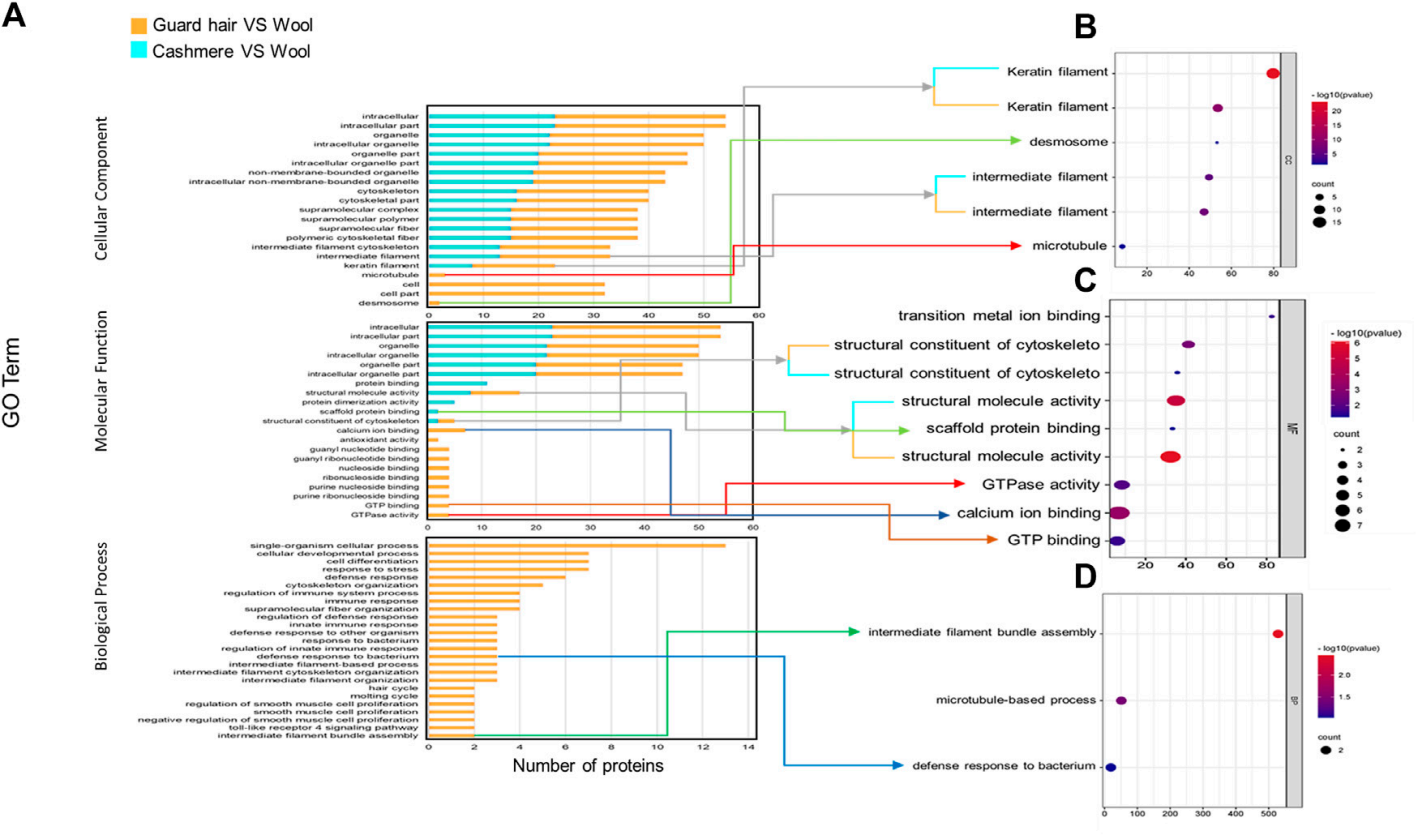
GO enrichment analysis for differentially expressed proteins (DEGs) from cashmere vs wool and guard hair vs wool. **(A)**. DEPs were enriched in three categories: biological process, molecular function, and cellular component. **(B)**. Direct GO terms in the cellular component category. **(C)**. Direct GO terms in the molecular function category. **(D)**. Direct GO terms in the biological process category.

**FIGURE 5:**
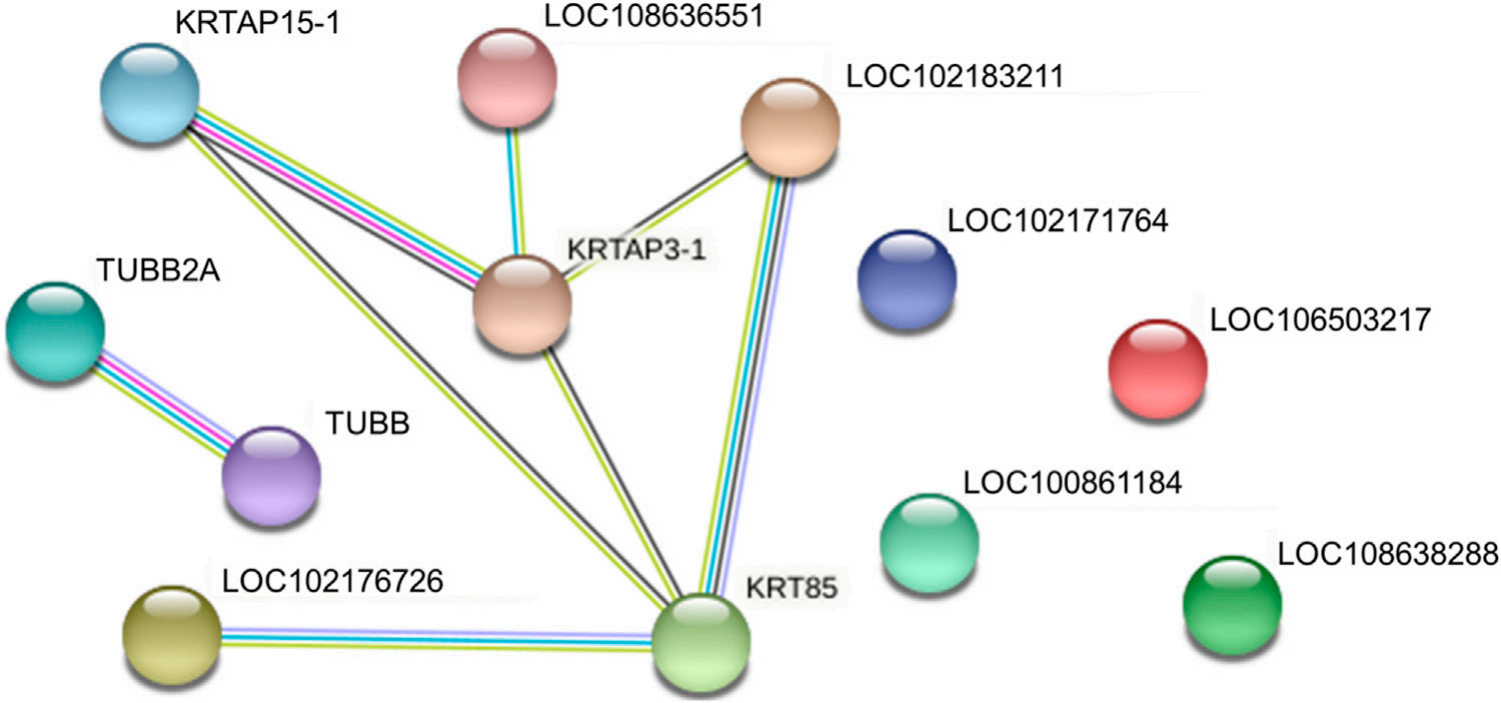
Protein‒protein interaction networks of identified differentially expressed proteins in cashmere, guard hair, and wool.

We identified proteins with significant abundance differences among each pairwise group, and fold change >2 or <0.5 and *p* < 0.05 were used as cutoff values to screen the proteins for further investigation. Volcano plots of differentially expressed proteins were prepared using the values of log(fold change) and log10 (*p* value). Interestingly, only 41 proteins were identified as being significantly different in cashmere and wool (Figure 3A); 54 proteins were identified as being significantly different when comparing wool and guard hair (Figure 3B). Remarkably, thirteen proteins were identified in fiber pairwise samples. The abundance of KRTAP15-1, KRTAP3-1, KRTAP9-2, LOC102183211, and TUBB2A are highest in cashmere, while the expression abundance of KRTAP15-1, LOC100861184, LOC102171764, LOC102176726, LOC106503217, LOC108636551, LOC108638288, and TUBB is highest in guard hair, and LOC102171764 is highest in wool (Figure 3C), emphasizing the importance of these thirteen proteins in determining hair characteristics associated with fiber diameter. To investigate the function of these different proteins (DEPs), we further performed a Gene Ontology (GO) analysis (Figure 4A). In the cell process category, the DEPs of the cashmere vs. wool group were enriched directly in keratin filaments and intermediate filaments, and the DEPs of the guard hair vs. wool group were enriched directly in keratin filaments, intermediate filaments, desmosomes, and microtubules (Figure 4B). In the molecular function category, the DEPs of the cashmere vs wool group were enriched directly in structural molecule activity, structural constituent of the cytoskeleton, and scaffold protein binding; the DEPs of the guard hair vs wool group were enriched directly in structural molecule activity, calcium ion binding, structural constituent of the cytoskeleton, GTP ase activity, transition metal ion binding, and GTP binding (Figure 4C). In the biological process category, the DEPs of the guard hair vs wool group were enriched directly in intermediate filament bundle assembly, microtubule-based process, and defense response to the bacterium (Figure 4D). PPI network analysis of DEPs in the STRING database was used to identify the interrelations of differentially expressed proteins among the hair fibers. A strong interaction was found with intermediate filaments, supramolecular fibers, and the cytoskeleton (Figure 5). The prediction of the protein interaction networks showed that KRTAP15-1, KRTAP3-1, KRT85, LOC108636551, LOC102183211, and LOC102176726 had pivotal roles in the network.

### Transcriptomic analysis using goat skin tissues

**FIGURE 6:**
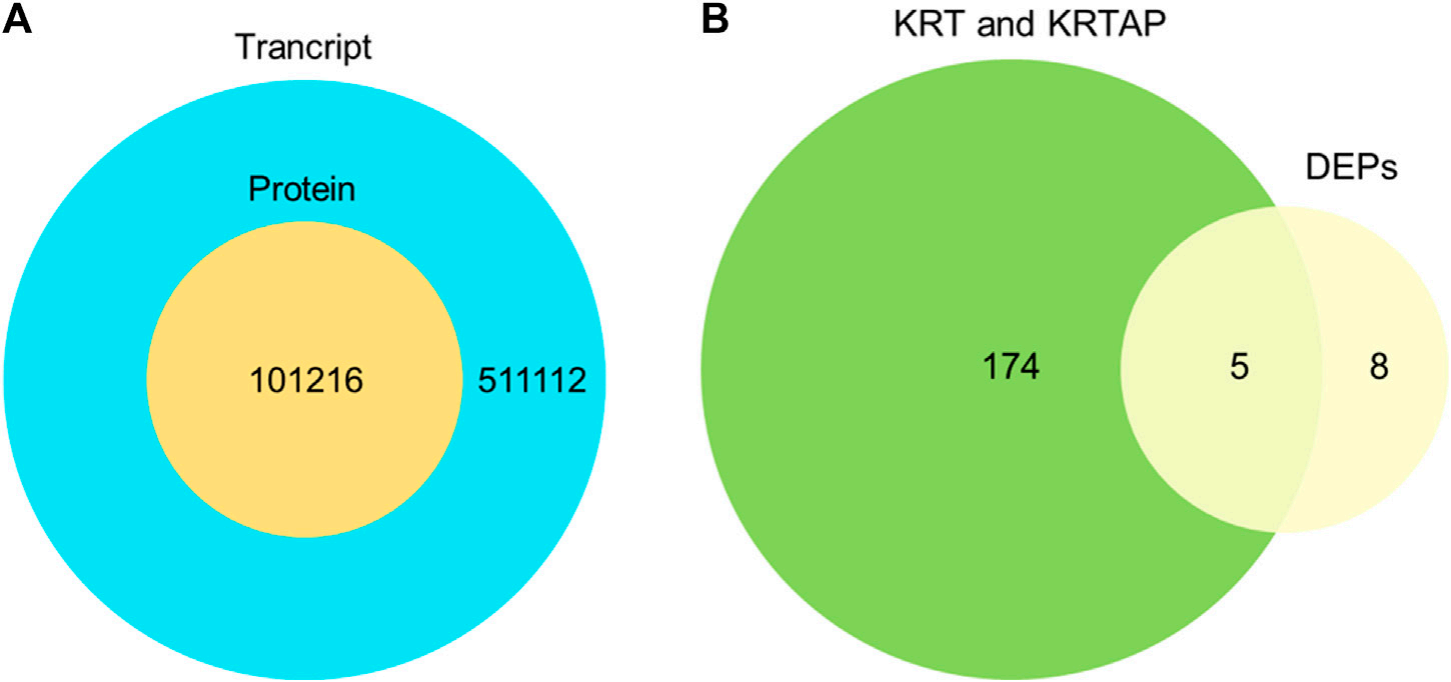
Comparison of the transcriptome and proteome. **(A)**. Overlap of the identified transcript and identified protein. **(B)**. Overlap of expressed genes of keratin and keratin-associated proteins and differentially expressed proteins.

To further validate the protein expression abundance patterns in goat skin, RNA-seq was conducted using skin tissues from cashmere goats (*n* = 12). RNA libraries for skin samples were constructed, and raw paired-end reads were generated using the Illumina HiSeq2000 sequencer. In total, 511,112 genes were expressed in the skins of cashmere goats. There were 121 keratins and 53 keratin-associated proteins (Figure 6A). Our transcriptomic and proteomic analyses showed that only five keratin/keratin-associated proteins overlapped in cashmere goats. These five genes were KRT85, KRTAP15-1, KRTAP3-1, KRTAP9-2, and LOC100861184 (Figure 6B). Importantly, KRTAP15-1 is related to fiber diameter (Niimura and Nei, 2006), highlighting its importance in the determination of fiber differences in goats.

### Multiple sequence alignment

**FIGURE 7:**
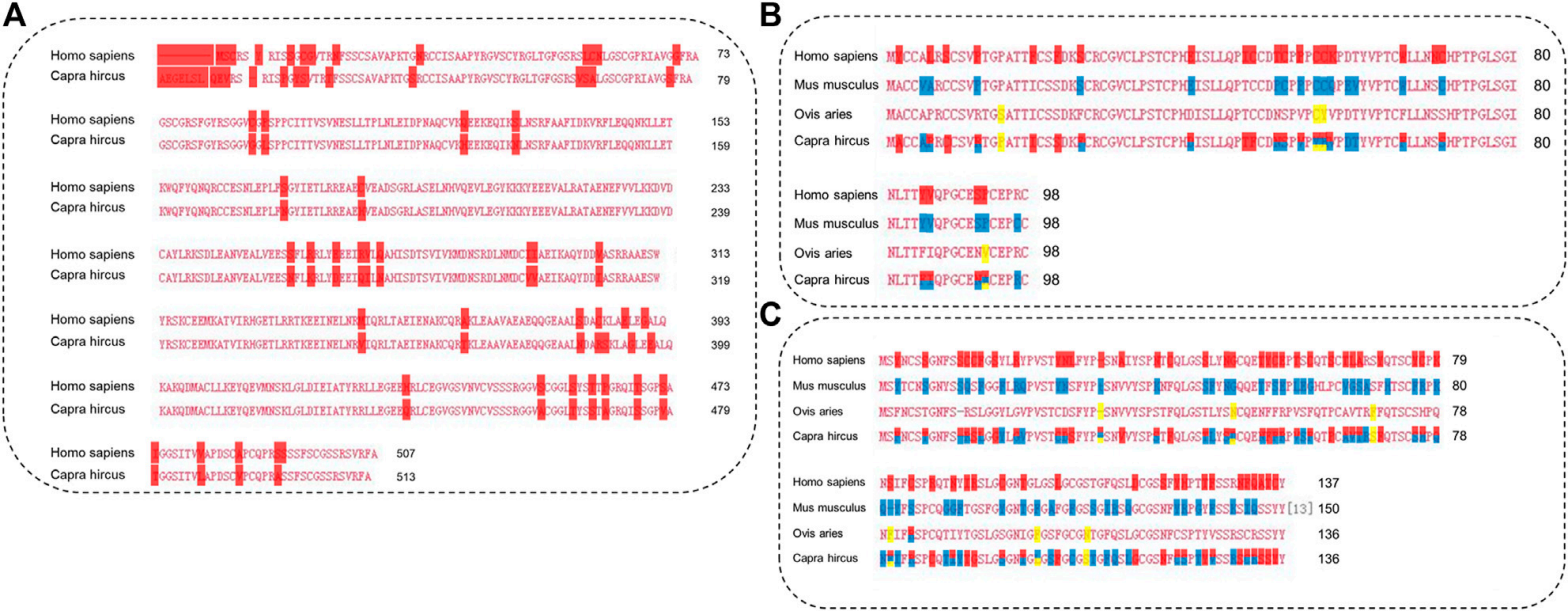
The amino acid sequences of proteins were analyzed by multiple sequence alignment. **(A)**. Multiple sequence alignment of KRT85. **(B)**. Multiple sequence alignment of KRTAP3-1. **(C)**. Multiple sequence alignment of KRTAP15-1.

**FIGURE 8:**
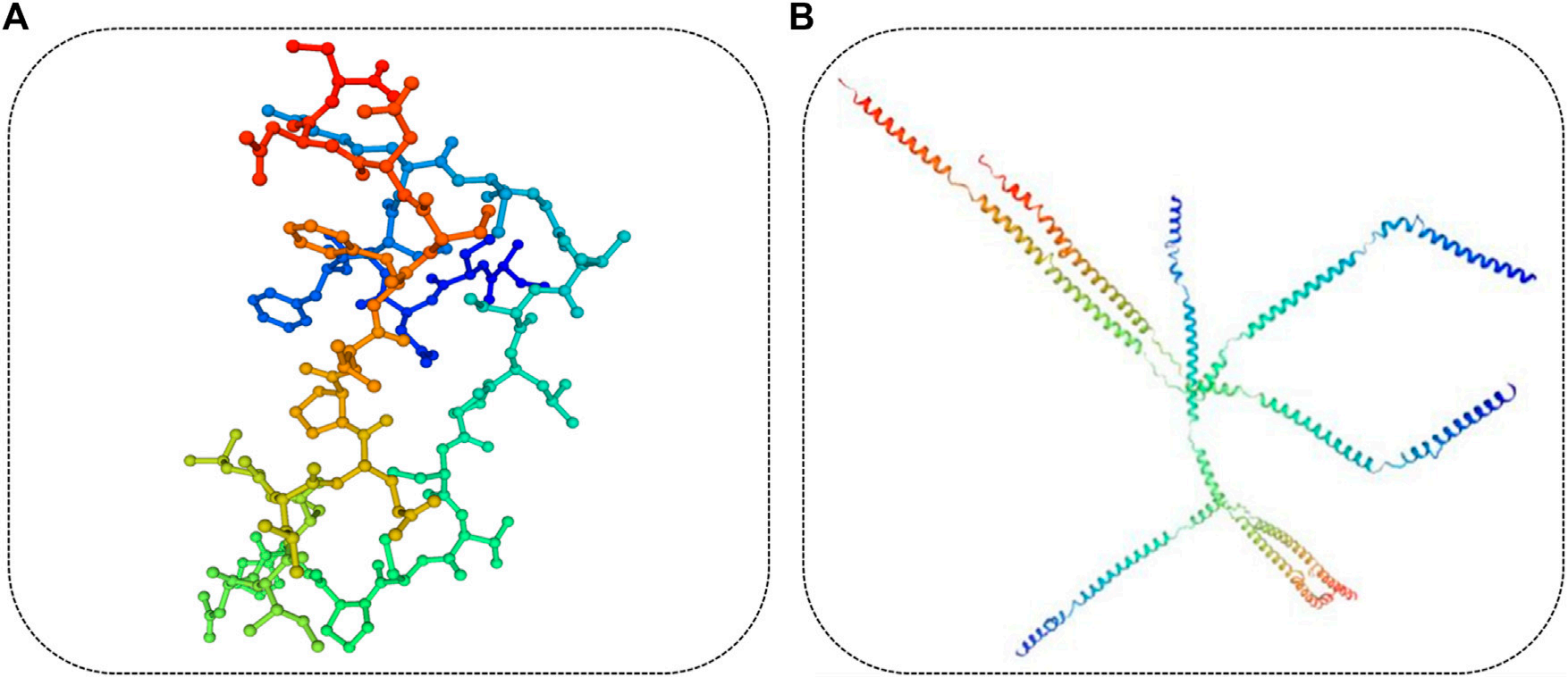
Three-dimensional structure of the protein. **(A)**. The three-dimensional structure of KRTAP3-1 was predicted based on the amino acid sequence. **(B)**. The three-dimensional structure of KRT85 was predicted based on the amino acid sequence.

The length of the amino acid sequence of KRT85 was 513 amino acids, KRTAP3-1 was 98 amino acids, and KRTAP15-1 was 136 amino acids. Importantly, the length of the KRTAP3-1 sequence was identical to that of *Ovis aries*, *Homo sapiens*, and *Mus musculus* obtained from NCBI/PROTEIN. The length of the KRTAP15-1 sequence was identical to that of *Ovis aries* obtained from NCBI/PROTEIN. Sequence alignment using NCBI/BLAST revealed that the amino acid sequence of KRT85 was 90% identical to that of *Homo sapiens*, the amino acid sequence of KRTAP3-1 was 77% identical to that of *Homo sapiens*, 80% and 96% identical to that of *Mus musculus*, *Ovis aries*, the amino acid sequence of KRTAP15-1 was 61% identical to that of *Homo sapiens*, and 65% and 96% identical to that of *Mus musculus* and *Ovis aries* (Figure 7). The red mark in Figure 7 represents the differentially expressed sequence of *Capra hircus* and *Homo sapiens*. The blue mark denotes the site difference in expression from that of *Mus musculus*, and the yellow mark represents the site with a difference from the sequence of *Ovis aries*. The three-dimensional structures of KRT85 and KRTAP3-1 were established by the comparative protein modeling program SWISS-MODEL (Figure 8).

## Discussion

Among domesticated animals, proteomics (along with other genetic techniques) is now regarded as a major promise for the development of animal science to meet the challenges of agriculture in the 21st century (Lippolis and Reinhardt, 2008). China is the world’s largest producer of cashmere fibers (Wu et al., 2020a). Cashmere goats thus play an important role in the development of China’s animal husbandry. Rapid developments in the textile industry mean that the requirements for fiber fineness are becoming increasingly strict. The diameter of wool fiber has important commercial value in fiber production because wool fiber will affect the performance of animals in the process of fabric production, so it becomes the goal of the animal breeding program. Accumulated historical demand and artificial selection of specific fiber types lead to the diversity of fleece types. For this reason, we attempted to form a comprehensive view of proteins involved in the composition of selected animal fibers (Li et al., 2018). To separate fiber proteins that are associated with fleece type, in this work, we performed traits and proteome-wide analysis of hair fibers with changed phenotypes in sheep and goats. It can be seen from the apparent structure that there are significant differences in the diameter of the three fibers. The scale structure of the fibers was observed by scanning electron microscopy. The scale height of cashmere and wool goats is different, the compactness is different, and the smoothness is also different. Structural differences are closely related to the mechanism of fiber formation.

Keratin (KRT) and keratin-associated proteins (KRTAP) are the main structural components of hair fibers (Li et al., 2021). As the principal structural proteins, they form the matrix between the keratin intermediate filaments in fiber and together they determine the quality of the hair fiber in the wool of sheep and cashmere of goats, as well as general hair in mammals (Hearle, 2000; Seki et al., 2011). To date, over 100 KRTAPs have been identified in mammals across a range (Gong et al., 2010; Khan et al., 2014; Gong et al., 2016), and 88 functional KRTAPs have been reported in human species (Rogers and Schweizer, 2005; Rogers et al., 2007; Rogers et al., 2008). Li studied Chinese Merino and Small-tail Han sheep, mohair Angora goats and cashmere goat and identified 173 proteins in sheep and goats (Li et al., 2018). We found that KRT and KRTAP accounted for the majority of detectable proteins in hair fibers by proteomic analysis. A total of 221 well-characterized proteins were found to be shared in sheep and goat fibers, including 46 keratins and 23 keratin-associated proteins. We revealed 38 (KRT24、KRT40、KRT85、KRTAP3-1、KAP、KRTAP9-2、KRTAP13-3、KRTAP15-1、KRTAP16-5、ASPRV1、BANF1、BMPR1B、CAPG、CALML5、DBI、FAM25A、TUBB、TUBB2A、TUBA4A、PSMD1、 PSAP、MGAT2、LOC102176726、LOC108638285、LOC102185436、LOC108638288、LOC102183211、LOC100861184、LOC108636551、LOC106503217、LOC102169411、LOC102179515、LOC108634682、LOC108636047、LOC102171395、LOC108636430、LOC102171764、LOC102175621) and 48(KRT1、KRT14、KRT17、KRT35、KRT75、KRT85、KRTAP3-1、KRTAP9-2、KRTAP15-1、BPIFB1、CTNNBIP1、DSG4、FABP3、GRN、GLRX、LPO、MYLPF 、OBP2、PKP1、S100A3、S100A14、TUBB、TUBB2A、TUBB4B、TCHH、TPM1、TPI1、YWHAG、LOC102176726、LOC102183766、LOC102177561、LOC108638288、LOC100861381、LOC102189437、LOC108635997、LOC102174594、LOC108636552、LOC102183211、LOC102176161、LOC108636551、LOC100861184、LOC108633164、LOC106503217、LOC106503204、LOC108637981、LOC102180495、LOC102171764、LOC100861174) proteins with abundance differences a cashmere, wool and guard fiber in sheep and goats, most of which were related to keratin filament, intermediate filament and structural molecule activity. Beta-tubulin (TUBB2A, TUBB2B, TUBB3, TUBB4A, TUBB) is important components of cytoskeleton which used to determine cell shape and movement (Romaniello et al., 2018). Additionally, proteins belong to the major transmembrane components, the desmogleins (DSG), were also identified in differentially expressed proteins. DSG is a cadherin type adhesion molecule in cells that is essential for cell types requiring high mechanical stress, such as skin keratinocytes and cardiomyocytes (Bazzi and Christiano, 2007). They were identified as differentially abundant proteins in sheep and goat hair fibers, indicating their importance in the composition and function of the hair fiber from different genotypes.

To validate the outcomes of HPLC‒MS/MS analysis at both the proteomic and transcriptomic levels, we performed RNA-seq using skin tissues to assess functional molecules that might be involved in the fiber differences between sheep and goats. Importantly, the KRTAP15-1 gene was reported as a strong candidate that affects wool yield, but it only had a trend of association with wool Mean fiber diameter (MFD) (Li et al., 2018). KRTAP15-1 and KRTAP3-1 are strongly associated with hair growth and are involved in the construction of hair, and they are more highly expressed in the anagen phase than in the catagen stage (Zhou et al., 2018; Zheng et al., 2019). KRT85 have significant effects on wool traits, it were significantly associated with wool crimps score, body size, and fiber diameter (*p* < 0.05) suggesting that these genes are important candidate genes for wool traits (Sulayman et al., 2018). KRT85, KRTAP15-1, and KRTAP3-1 might explain the phenotypic differences in cashmere and guard hair and suggest that they play a role in fiber development. This study provides a useful reference for further understanding the relationship between KRT/KRTAP and cashmere fineness, and it will contribute to knowledge regarding the development of cashmere traits.

## Conclusion

Through label-free proteomics analysis, we identified 226 proteins in hair fiber between sheep and goats. Proposed HPLC‒MS/MS methods were used to identify novel differentially abundant proteins. Our comprehensive proteome maps of different fibers from sheep and goats, two major fiber-producing species, enabled us to determine many proteins of different fiber types. We reveal proteins, such as KRT85, KRTAP15-1, KRTAP3-1, KRTAP9-2, and LOC100861184, which may be related to fiber diameter and facilitated the understanding of the composition and function of hair fibers in mammals.

## Data Availability

The mass spectrometry proteomics data have been deposited to the ProteomeXchange Consortium (http://proteomecentral.proteomexchange.org) via the iProX partner with the dataset identifier http://proteomecentral.proteomexchange.org/cgi/GetDataset?ID=PXD036685 (PXD036685).
